# Fear of Reinjury Following Surgical and Nonsurgical Management of Anterior Cruciate Ligament Injury: An Exploratory Analysis of the NACOX Multicenter Longitudinal Cohort Study

**DOI:** 10.1093/ptj/pzab273

**Published:** 2021-12-22

**Authors:** Stephanie Filbay, Joanna Kvist

**Affiliations:** Centre for Health Exercise and Sports Medicine, Department of Physiotherapy, University of Melbourne, Victoria, Australia; Department of Health, Medicine and Caring Sciences, Division of Prevention, Rehabilitation and Community Medicine, Unit of Physiotherapy, Linköping University, Linköping, Sweden; Center for Medical Image Science and Visualization (CMIV), Department of Health, Medicine and Caring Sciences, Linköping University, Linköping, Sweden; Stockholm Sports Trauma Research Center, Department of Molecular Medicine & Surgery, Karolinska Institute, Stockholm, Sweden

**Keywords:** Anterior Cruciate Ligament Reconstruction, Decision Making: Clinical, Knee Function, Orthopedics, Rehabilitation, Self-Efficacy

## Abstract

**Objectives:**

The purpose of this study was to compare fear and certainty of reinjury between follow-up time points and treatment groups (no anterior cruciate ligament [ACL] reconstruction [no ACLR], pre-ACLR, post-ACLR) and to identify prognostic factors for fear of reinjury at 3 and 12 months following injury or ACLR.

**Methods:**

An exploratory analysis of the Natural Corollaries and Recovery After ACL-injury multicenter longitudinal cohort study was conducted. Patients (n = 275) with primary ACL injury and 15 to 40 years of age received usual care (initial physical therapist–supervised rehabilitation, before considering ACLR). Fear of reinjury (as measured with the Anterior Cruciate Ligament Quality of Life instrument [ACL-QOL] item 31 and the Anterior Cruciate Ligament Return to Sport After Injury instrument [ACL-RSI] item 9) and certainty of reinjury (as measured with the Knee Self-Efficacy Scale item D2) were evaluated at baseline and at 3, 6, and 12 months following ACL injury or ACLR. Comparisons were performed with linear mixed models. Linear regression assessed potential prognostic factors (age, sex, preinjury activity, baseline knee function, baseline general self-efficacy, and expected recovery time) for fear of reinjury (ACL-QOL item 31) at the 3- and 12-month follow-up assessments.

**Results:**

Fear of reinjury was common regardless of ACL treatment. Fear of reinjury decreased between 3 and 6 months and 3 and 12 months (mean difference: ACL-QOL = 9 [95% CI = 2 to 15]; ACL-RSI = 21 [95% CI = 13 to 28]) after injury. This improvement was not observed in patients who later underwent ACLR, who reported worse fear of reinjury at 3 months (ACL-QOL = 10 [95% CI = 3 to 18]) and at 12 months (ACL-RSI = 22 [95% CI = 2 to 42]) postinjury compared with those who did not proceed to ACLR. Following ACLR, fear of reinjury decreased between the 3- and 12-month follow-up assessments (ACL-QOL = 10 [95% CI = 4 to 16]; ACL-RSI = 12 [95% CI = 5 to 19]). Greater baseline general self-efficacy was associated with reduced fear of reinjury at 12 months after injury (adjusted coefficient = 1.7 [95% CI = 0.0 to 3.5]). Female sex was related to more fear of reinjury 3 months after ACLR (−14.5 [95% CI = −25.9 to −3.1]), and better baseline knee function was related to reduced fear of reinjury 12 months after ACLR (0.3 [95% CI = 0.0 to 0.7]).

**Conclusion:**

People who had ACLR reported worse fear of reinjury before surgery than those who did not proceed to ACLR. Different prognostic factors for fear of reinjury were identified in people treated with ACLR and those treated with rehabilitation alone.

**Impact:**

Fear of reinjury is a concern following ACL injury. Clinicians should evaluate and address reinjury fears. These results may assist in identifying individuals at risk of fear of reinjury following surgical and nonsurgical management of ACL injury.

## Introduction

Anterior cruciate ligament (ACL) injury can have serious physical and psychological health consequences.^1–9^ Fear of reinjury is the most common psychological barrier to rehabilitation progression following ACL injury^10^ and is associated with fear avoidance behaviors and ceasing participation in sport and recreational activities.^11–14^ A recent review found that 20% to 45% of individuals reported that fear of reinjury was the reason they did not return to sport after ACL injury.^10^ Not returning to sport and high fear of reinjury after ACL injury are associated with poor long-term quality of life.^11^^,^^12^^,^^15^

There is ongoing debate about whether ACL reconstruction (ACLR) is the gold standard treatment for ACL injury despite the best available evidence demonstrating similar outcomes (including pain, symptoms, function, quality of life, and return-to-sport rates) following ACLR and nonsurgical management with physical therapist–supervised rehabilitation.^1^^,^^16^^,^^17^ However, the relationship between ACL management approach and fear of reinjury is not yet understood. The fear of undergoing ACL surgery and postoperative recovery again may be an important contributor to fear of reinjury following ACLR.^18^ Additionally, it is possible that the physical and psychological experience of ACLR contributes to persistent reinjury fears. On the other hand, undergoing ACLR may assist some patients in alleviating fear of reinjury.^19^ To our knowledge, only 1 study has evaluated fear of reinjury (using a measure of kinesiophobia) in patients treated with rehabilitation alone and made comparisons between management strategies.^20^ Tengman et al found that more than 20 years following ACL injury, people treated with ACLR reported similarly high levels of fear of reinjury compared with those treated with physical therapy–based rehabilitation.^20^ Few studies have investigated fear of reinjury in individuals with ACL injury managed nonsurgically, and further research is needed to determine if fear of reinjury differs between individuals treated with surgery and individuals treated with rehabilitation alone.

Fear of reinjury (kinesiophobia) may decrease with time after ACLR. Hartigan et al found that fear of reinjury (kinesiophobia) improved for all patients between preoperative and 6 and 12 months after ACLR.^19^ Two studies found that within 12 weeks of ACLR, fear of reinjury (kinesiophobia) improved but not to a meaningful degree.^21^^,^^22^ Theunissen et al found the number of patients with high fear of reinjury (kinesiophobia) reduced between 3 and 12 months after ACLR.^23^ Additionally, fear of reinjury improved to a greater degree after ACLR in patients with worse preoperative knee function and patient-reported measures.^19^ To the authors’ knowledge, no study has evaluated changes in fear of reinjury over time after nonsurgically treated ACL injury. In addition, no study—to our knowledge—has compared this between groups treated surgically and groups not treated surgically.

Most studies investigating fear of reinjury following ACL injury assessed the relationship between fear of reinjury and return to sport or the correlation between fear of reinjury and other knee measures at a single point in time. Few studies have investigated prognostic factors for increased fear of reinjury after ACL injury. Theunissen et al investigated preoperative characteristics (age, sex, body mass index, injury-to-surgery time, preoperative knee pain, and preoperative self-reported knee function) as potential predictors of fear of reinjury at 3 months after ACLR.^23^ This study identified prolonged injury-to-surgery time, high preoperative pain level, male sex, and low body mass index as predictors of greater fear of reinjury (assessed using a measure of kinesiophobia) at 3 months after ACLR.^23^ However, little is known about other potential prognostic factors for increased fear of reinjury, and prognostic factors have not been investigated in patients treated nonsurgically (with physical therapist–supervised rehabilitation). Considering fear of reinjury is potentially modifiable, an improved understanding of prognostic factors for fear of reinjury is needed.^1^ Additionally, comparison of prognostic factors for fear of reinjury between patients treated surgically and those treated nonsurgically could assist in identifying individuals better suited to either management strategy.

Studies that have aimed to assess “fear of reinjury” in individuals with ACL injury have used a variety of questionnaires. These questionnaires include single items addressing fear of reinjury and were designed to measure different constructs, including fear of movement/kinesiophobia in people with chronic pain,^24^ psychological readiness and impact of returning to sport after ACL injury (ie, the Anterior Cruciate Ligament Return to Sport After Injury Instrument [ACL-RSI]),^25^ and fear and avoidance beliefs about physical activity and work in people with low back pain.^26^ Most studies investigating fear of reinjury after ACL injury have used the Tampa Scale for Kinesiophobia,^24^ which was not designed for this purpose. Kinesiophobia has been defined as “an excessive, irrational, and debilitating fear of physical movement and activity resulting from a feeling of vulnerability to painful injury or reinjury.^27^” This construct may not capture elements of fear of reinjury specific to ACL-injured individuals. Meierbachtol et al found that the ACL-RSI score better identified the intensity of fear for individual fear-evoking tasks or situations and for fear of reinjury than the Tampa Scale for Kinesiophobia.^28^ Because there is no available instrument designed to evaluate fear of reinjury that is appropriate for use in ACL-injured individuals, single items that assess fear of reinjury extracted from questionnaires designed for use in populations with ACL injury may provide a useful means to investigate fear of reinjury in this population. Additional insights may be gained by using multiple items (eg, items assessing fear of injury, certainty of reinjury, fear of contact sports) and assessing for similarities and differences between items.

This study will generate prognostic information that may be used to identify individuals with ACL injury and at risk of experiencing fear of reinjury following ACLR or management with rehabilitation alone. Additionally, this study, using a prospective longitudinal cohort, will address knowledge gaps, including changes in fear of reinjury over time and comparisons in fear of reinjury between people treated with ACLR and those treated with rehabilitation alone. This research could inform preventative strategies and early interventions to optimize long-term outcomes after ACL injury. The 2 aims of this study were to compare fear and certainty of reinjury between follow-up time points (3-, 6-, and 12-month follow-up assessments) and ACL treatment groups (no ACLR [ie, rehabilitation alone], pre-ACLR, post-ACLR) and to identify prognostic factors (potential prognostic factors explored include age at injury, sex, preinjury activity level, baseline knee function, general self-efficacy, and expectations for recovery) for fear of reinjury at 3 and 12 months following ACL injury managed with ACLR or rehabilitation alone. We hypothesize that fear of reinjury will improve over time in all treatment groups, that fear of reinjury will be greater after ACLR compared with treatment with rehabilitation alone, and that prognostic factors for fear of reinjury will differ depending on management approach.

## Methods

This is an exploratory analysis of data from the Natural Corollaries and Recovery After ACL-injury (NACOX) Study (trial registration number NCT02931084), a multicenter longitudinal prognostic cohort study designed to investigate the effects of ACL injury managed with usual care in Sweden.^29^ This study was approved by the Swedish Ethical Review Authority (Dnr 2016/44-31 and 2017/221–32).

Between May 2016 and October 2018, patients who presented to 1 of 7 health care clinics across Sweden (including both public and private clinics) who sustained an ACL injury no more than 6 weeks previously and were aged between 15 and 40 years at the time of injury were invited to participate in the NACOX Study. ACL ruptures were diagnosed by an orthopedic surgeon. Patients were ineligible if they had a previous ACL injury to the same knee, serious concomitant injury (eg, fracture that required specific treatment, posterior cruciate ligament rupture), were unable to understand written or spoken Swedish, or had cognitive impairments or other illnesses or injuries that impaired function (eg, fibromyalgia, rheumatic diseases, or other diagnoses associated with chronic pain). Patients provided informed consent prior to study enrollment.

Following ACL injury, individuals followed the usual course of treatment at recruiting centers, which was initial physical therapist–supervised rehabilitation for approximately 3 months, before a follow-up with an orthopedic surgeon to decide on undergoing ACLR or continuing with rehabilitation alone. Some patients and clinicians made a shared decision to undergo early ACLR within 3 months from injury.^30^ The main reasons for nonsurgical treatment were “no knee instability” and “no problems with knee function.”^30^ The main reason for undergoing early ACLR (within 31 days of injury) was “high activity demands.”^30^ In the subacute phase (32 days to 5 months after injury), “instability/giving way” and high-activity demands were the main reasons for undergoing ACLR, and a later ACLR decision was most often due to instability/giving way.^30^

**Figure 1:**
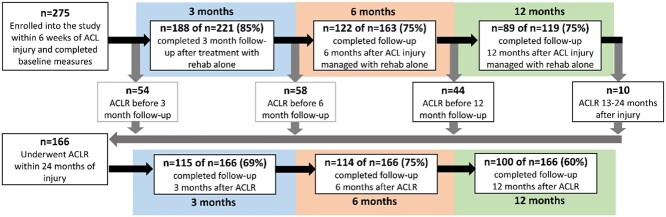
Participant flow and response rate. ACL = anterior cruciate ligament; ACLR = ACL reconstruction; rehab = rehabilitation.

Questionnaires were sent to the participants via a short mobile phone message or email at various times up to 3 years after injury or ACLR.^29^ For this exploratory analysis, we included data from baseline (within 6 weeks of injury) and follow-up at 3, 6, and 12 months after injury or ACLR. We included these time points to capture potential change in fear of reinjury throughout the rehabilitation process and compare this between treatment strategies. We included time points up to 12 months after injury/ACLR when patients are typically considered ready to return to full participation in sports.^1^ In total, 275 participants were included in the NACOX Study, and all were included in this exploratory analysis. At 12 months following ACL injury, 119 participants (43%) had not undergone ACLR, and 156 (57%) had an ACLR within 3 months (n = 54), 3 to 6 months (n = 58), or 6 to 12 months (n = 44) after ACL injury (Fig. 1). An additional 10 participants underwent ACLR 12 to 24 months after ACL injury, resulting in 166 individuals with ACLR being sent follow-up questionnaires. The number of participants treated with ACLR and those not treated with ACLR at each follow-up time point and response rate are presented in Figure 1.

## Treatment Groups

To address the first study aim, outcomes were assessed at the 3-, 6-, and 12-month follow-up assessments, stratified into 3 treatment groups: no ACLR, managed with rehabilitation alone and did not undergo ACLR within 24 months of injury (3 months: n = 76; 6 months: n = 68; 12 months: n = 79); pre-ACLR, managed with rehabilitation alone at the time of follow-up but underwent ACLR within 24 months of injury (3 months: n = 112; 6 months: n = 54; 12 months: n = 10); and post-ACLR, follow-up completed at 3, 6, or 12 months following ACLR (3 months: n = 115; 6 months: n = 114; 12 months: n = 100) (Fig. 1). To address the second study aim, patients were classified according to their ACL treatment status at the time of the 3- and 12-month follow-up assessments into “rehabilitation” and post-ACLR (classification outlined above) groups. The rehabilitation group comprised all participants who had not had ACLR at the time of follow-up (3 months: n = 188; 12 months: n = 89) (Fig. 1).

### Outcomes

The primary outcome is fear of reinjury assessed with item 31 of the Anterior Cruciate Ligament Quality of Life instrument (ACL-QOL).^31^ The item asks participants “How fearful are you of reinjuring your knee?” with responses ranging from 0 (extremely fearful) to 100 (no fear at all) with 10-point increments. Secondary outcomes include “fear of reinjury during sport” assessed with item 9 of the ACL-RSI (“Are you afraid of accidentally injuring your knee by playing your sport?” with response options ranging from 0 [extremely afraid] to 100 [not at all afraid])^25^^,^^32^ and “certainty of knee reinjury” assessed with item D2 from the Knee Self-Efficacy Scale (K-SES) for ACL injury (“How certain are you that you would not suffer any new injuries to your knee?” with responses on an 11-grade Likert scale ranging from 0 [not at all certain] to 10 [very certain]).^33^

Because there is no reliable and valid instrument available for evaluating fear of reinjury in individuals with ACL injury, we selected a combination of items extracted from measures of knee-related quality of life (ACL-QOL),^31^ psychological impact of returning to sport after ACLR (ACL-RSI),^25^ and knee self-efficacy (K-SES) found to be valid and reliable for use in populations with ACL injury.^34–36^ We used a combination of items to allow exploration of similarities and differences between these items and to generate information that may be used to inform the development of an instrument to assess fear of reinjury after ACL injury.

### Potential Prognostic Factors

The 6 variables evaluated as potential prognostic factors for fear of reinjury at 3 and 12 months were collected within 6 weeks of ACL injury. Potential prognostic factors for fear of reinjury were selected based on knowledge of the literature (ie, a potential relationship or a knowledge gap^23^) and clinical reasoning and included age at injury, sex, preinjury activity level, baseline knee function, general self-efficacy, and expectations for recovery.

Preinjury activity level was evaluated with the Tegner Activity Scale, an 11-grade scale ranging from level 0 (no participation in physical activity due to knee problems) to level 10 (competing in elite football).^37^ Scores of 7 to 10 can only be achieved if the person participates in recreational or competitive sports with high functional knee demands. We hypothesized that people who took part in sports with high functional knee demands could experience greater fear of reinjury than those who took part in sports with lower knee demands. Therefore, for the analysis the Tegner Activity Scale was converted to a binary score (lower activity level = 0–6 vs higher activity level = 7–10). This classification is commonly used to assess participation in competitive sports because a score of ≤6 excludes participation in competitive sports. Baseline knee function was assessed with the Single Assessment Numeric Evaluation (SANE) rating (“On a scale from 0 to 100 where 100 is perceived as best, how would you rate your knee today?”).^38^ The SANE has a moderate to strong positive correlation with the International Knee Documentation Committee (IKDC), the modified Cincinnati Knee Rating System, and the Lysholm Scale in patients treated with ACLR or knee arthroscopy.^38^^,^^39^ The SANE minimizes participant burden and allows the respondent to consider what matters most to them when providing an overall knee rating rather than other measures of subjective knee function that assess a range of constructs (eg, knee symptoms, pain, confidence) and weight items equally in an overall composite score. We decided to use the SANE rather than the IKDC in this study because the IKDC assesses knee function in relation to sport participation, and sport participation is advised against within 6 months of ACL injury/surgery.

The Swedish version of the General Self Efficacy Scale assessed self-efficacy. A higher score across 10 items (maximum score = 40) indicates greater self-efficacy.^40^^,^^41^ Expectations for recovery were evaluated with a single item that is valid for use in a variety of populations^42^: “When do you think your knee will be recovered to the same level as before the injury?” (response options: within 1 week, within 1 month, within 6 months, within 12 months, more than 12 months, my knee will never recover). This was converted to a binary variable for analysis to enable evaluation of the effect of a more optimistic expectation (expected recovery at ≤12 months), which we hypothesized may be related to reduced fear of reinjury compared with a less optimistic expectation (more than 12 months/my knee will never recover).

### Statistical Analysis

Fear of reinjury (assessed using ACL-QOL item 31), fear of reinjury during sport (assessed using ACL-RSI item 9), and certainty of knee reinjury (assessed using K-SES item D2) are presented as mean scores and SDs at baseline (within 6 weeks of injury) and at 3, 6, and 12 months following ACL injury managed with rehabilitation alone and at 3, 6, and 12 months following ACLR. Available data at each time point (the ACL-QOL was not assessed at 6 months, and the ACL-RSI was not assessed at baseline) are depicted in online graphs.

Linear mixed models, using maximum-likelihood estimation with the assumption of unstructured covariance, were used to estimate fear of reinjury (ACL-QOL item 31), fear of reinjury during sport (ACL-RSI item 9), and certainty of reinjury (K-SES item D2) at the 3-, 6-, and 12-month follow-up assessments in the no ACLR, pre-ACLR, and post-ACLR groups. Each model included the fixed effect of time, group, and time × group interaction. The difference in outcomes within groups at the 3- and 6-month follow-up assessments and at the 3- and 12-month follow-up assessments was assessed using a linear mixed model pairwise comparison based on estimated marginal means using the Bonferroni adjustment to account for multiple comparisons. Outcomes assessed at the 3-, 6-, and 12-month follow-up assessments were compared between the no ACLR and pre-ACLR groups as well as the no-ACLR and post-ACLR groups at each time point.

Linear regression assessed potential prognostic factors for fear of reinjury (assessed using ACL-QOL item 31) at 3 and 12 months following ACL injury managed with rehabilitation alone (ie, the rehabilitation group) and at 3 and 12 months following ACLR (ie, the post-ACLR group). Underlying assumptions for linear regression were assessed and met, including linearity between independent and dependent variables, multivariate normality, multicollinearity, autocorrelation, and homoscedasticity of residuals. Crude and adjusted effect estimates are reported in terms of regression coefficients, and 95% CIs present the estimated uncertainty. Discrimination is quantified with a coefficient of determination (*R*^2^). Because this is a prognostic cohort study not concerned with causation, adjustment for confounding factors was not required.^43^

**Table 1:** Participant Baseline Characteristics^a^

**Characteristic**	**All Participants (n = 275)**	**Patients Who Received Rehabilitation Alone**		**Patients Who ReceivedACLR + Rehabilitation** **(n = 166)**
		**3-mo FU** **(n = 221)**	**12-mo FU** **(n = 119)**	
Age, mean (SD), y	25 (7)	26 (7)	27 (7)	24 (7)
Female sex	143 (52)	117 (53)	54 (45)	94 (57)
BMI, mean (SD), kg/m^2^	24 (3)	24 (3)	25 (4)	24 (3)
Preinjury Tegner Activity Scale score*^b^*				
0–6: lower activity level	99 (36)	94 (43)	58 (49)	44 (27)
7–10: higher activity level	176 (64)	127 (58)	61 (51)	122 (74)
Preinjury IKDC activity level				
I: pivoting and contact	153 (56)	112 (51)	52 (44)	106 (64)
II: pivoting and non-contact	39 (14)	32 (14)	19 (16)	22 (13)
III: neither pivoting nor contact	83 (30)	77 (35)	48 (40)	38 (23)
Baseline knee function, SANE score, mean (SD)	39 (20)	39 (20)	41 (20)	38 (20)
General Self-Efficacy Scale score, mean (SD)	32 (4)	32 (4)	32 (4)	32 (5)
Expectation for recovery				
≤1 mo	30 (11)	27 (13)	19 (16)	12 (7)
1–6 mo	102 (38)	90 (42)	60 (51)	46 (28)
6–12 mo	93 (34)	62 (29)	22 (19)	72 (44)
>12 mo	27 (10)	21 (10)	8 (7)	21 (13)
Never	18 (7)	16 (7)	9 (8)	11 (7)

^*a*^Data are reported as number (percentage) of participants unless otherwise indicated. Baseline characteristics are reported separately for patients who received rehabilitation alone at 3 months following injury (3-mo FU) and those who remained non–surgically treated at 12 months after ACL injury (12-mo FU). ACL = anterior cruciate ligament; ACLR = ACL reconstruction; BMI = body mass index; IKDC = International Knee Documentation Committee^44^; SANE = Single Assessment Numeric Evaluation. Deviations in total counts for specific variables compared with the n = reported in each column heading is due to missing data.

^*b*^Preinjury activity level was evaluated with the Tegner Activity Scale, an 11-grade scale ranging from level 0 (no participation in physical activity because of knee problems) to level 10 (competing in elite football); scores of 7 to 10 can be achieved only if the person participates in recreational or competitive sports with highly functional knee demands.

### Missing Data

Incomplete data for potential prognostic factors: preinjury activity level (n = 1, 0.4%), baseline knee function (n = 9, 3%), baseline general self-efficacy (n = 3, 1%), and expected recovery (n = 5, 2%) were assessed for systematic patterns and monotonicity and assumed to be missing at random. Multiple imputation using 40 iterations was performed to account for missing values using the Markov Chain Monte Carlo technique. Consistency between imputation iterations and convergence between complete data and imputed data were assessed by comparing proportions and regression coefficients. Missing data were not imputed for the primary outcome (ACL-QOL item 31: 11% had missing data at 3 months and 8% had missing data at 12 months in the rehabilitation group; 9% had missing data at 3 months and 8% had missing data at 12 months following ACLR). All analyses were performed using IBM SPSS Statistics for Windows, Version 27.0 (IBM Corp., Armonk, NY, USA).

### Role of the Funder

The funders played no role in the design, conduct, or reporting of this study.

## Results

### Participant Characteristics

The mean age of the participants was 25 (SD = 7) years old, and 52% were women (Tab. 1). Among patients who were treated with ACLR, 74% were participating in recreational or competitive sport with highly functional knee demands before ACL injury compared with 51% of patients who were treated with rehabilitation alone at the 12-month follow-up assessment (Tab. 1). The majority expected that their knee would recover within 12 months (n = 225, 83%), and 7% (n = 18) expected that their knee would never recover. Twenty-one patients (8%) had a previous history of contralateral ACL injury, of whom 14 had undergone ACLR on their contralateral knee.

### Fear and Certainty of Reinjury

#### Fear of Reinjury (ACL-QOL Item 31)

**Figure 2:**
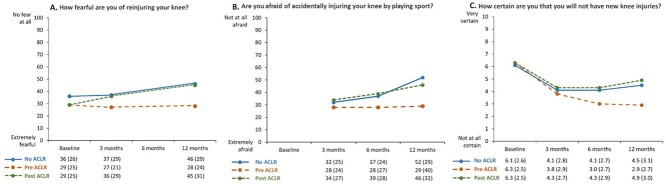
Fear of reinjury assessed with Anterior Cruciate Ligament Quality of Life instrument (ACL-QOL) item 31 (A), fear of reinjury during sport assessed with Anterior Cruciate Ligament Return to Sport After Injury instrument (ACL-RSI) item 9 (B), and certainty of knee reinjury assessed with the Knee Self-Efficacy Scale (K-SES) item D2 (C), stratified by anterior cruciate ligament (ACL) treatment. Data represent mean (SD). Baseline represents assessment within 6 weeks of ACL injury. Data from 3, 6, and 12 months represent time since ACL injury (No ACLR and Pre ACLR) or time since ACL reconstruction (ACLR) (Post ACLR). No ACLR = management with rehabilitation alone (patient did not undergo ACLR within 24 months of injury); Pre ACLR = management with rehabilitation alone at the time of follow-up but patient underwent ACLR within 24 months of injury; Post ACLR = follow-up was completed at 3, 6, or 12 months following ACLR.

On average, fear of reinjury scores (ACL-QOL item 31) were low (indicating high levels of fear of reinjury) at all time points (Fig. 2A). Fear of reinjury decreased by an estimated 9 points (95% CI = 2 to 15; *P* = .01) between the 3- and 12-month follow-up assessments for the patients who had no ACLR within 24 months of injury (Tab. 2). Post-ACLR, fear of reinjury decreased by an estimated 10 points (95% CI = 4 to 16; *P* = .001) between the 3- and 12-month follow-up assessments. Patients who underwent an ACLR after follow-up (pre-ACLR group) reported greater fear of reinjury at 3 months (mean difference 10 points [95% CI = 3 to 18]; *P* = .009) following ACL injury than patients who did not undergo ACLR within 24 months of injury (no ACLR group) (Fig. 2A; Tab. 2).

**Table 2:** Fear and Certainty of Reinjury: Estimated Marginal Means and 95% CIs and Comparisons of Time Points and Treatment Groups^a^

	**Estimated Marginal Mean (95% CI) for:**							
**ACLR** ^***b***^	**Fear of Reinjury** **(ACL-QOL Item 31)**		**Fear of Reinjury During Sport (ACL-RSI Item 9)**			**Certainty of Reinjury** **(K-SES Item D2)**		
	**3 mo**	**12 mo**	**3 mo**	**6 mo**	**12 mo**	**3 mo**	**6 mo**	**12 mo**
No ACLR	37 (31 to 43)	45*^c^* (39 to 52)	31 (26 to 37)	38*^c^* (32 to 43)	52*^c^* (45 to 58)	4.1 (3.5 to 4.6)	4.0 (3.4 to 4.6)	4.4 (3.7 to 5.1)
Pre-ACLR	27*^d^* (21 to 32)	26 (8 to 45)	27 (22 to 32)	31 (24 to 38)	30*^d^* (11 to 48)	3.8 (3.2 to 4.4)	3.3 (2.5 to 4.1)	3.1 (1.2 to 4.9)
Post-ACLR	35 (30 to 41)	45*^c^* (39 to 51)	33 (28 to 38)	38 (33 to 43)	45*^c^* (39 to 51)	4.1 (3.6 to 4.6)	4.3 (3.8 to 4.9)	4.9*^c^* (4.3 to 5.5)

^*a*^ACL-QOL = Anterior Cruciate Ligament Quality of Life instrument; ACLR = anterior cruciate ligament (ACL) reconstruction; ACL-RSI = Anterior Cruciate Ligament Return to Sport After Injury instrument; K-SES = Knee Self-Efficacy Scale.

^*b*^No ACLR represents management with rehabilitation alone (patient did not undergo ACLR within 24 months of injury) Pre-ACLR represents management with rehabilitation alone at the time of follow-up, but patient underwent ACLR within 24 mo of injury. Post-ACLR represents follow-up completed at 3, 6, or 12 months following ACLR.

^*c*^The marginal mean was different from that at the 3-month follow-up (*P* ≤ .05).

^*d*^The marginal mean was different from the no ACLR group (*P* ≤ .05).

#### Fear of Reinjury During Sport (ACL RSI Item 9)

Overall, fear of reinjury during sport (ACL-RSI item 9) was high at 3, 6, and 12 months of follow-up irrespective of management strategy (Fig. 2B). For patients who had no ACLR within 24 months of injury, fear of reinjury was reduced by an estimated 7 points (95% CI =1 to 13; *P* = .03) between the 3- and 6-month follow-up assessments and 21 points (95% CI = 13 to 28; *P* < .001) between the 3- and 12-month follow-up assessments (Tab. 2). Post-ACLR, fear of reinjury was reduced by an estimated 12 points (95% CI = 5 to 19; *P* < .001) between the 3- and 12-month follow-up assessments. Patients who underwent ACLR after the 24-month follow-up assessment (pre-ACLR group) reported greater fear of reinjury at 12 months after injury (22 points [95% CI = 2 to 42]; *P* = .03) than patients who did not undergo ACLR within 24 months of injury (no ACLR group) (Tab. 2).

#### Certainty of Reinjury (K-SES Item D2)

Overall, certainty of not having a new knee reinjury (assessed using K-SES item D2) was low at the 3-, 6-, and 12-month follow-up assessments, irrespective of management strategy (Fig. 2C). Post-ACLR, patients became more certain that they would not reinjure their knee between 3 and 12 months after ACLR (0.79 [95% CI = 0.01 to 0.16]; *P* = .01). The linear mixed model showed no differences in certainty of reinjury between treatment groups (Tab. 2).

### Prognostic Factors for Fear of Reinjury at 3 and 12 Months After ACL Injury Managed With Rehabilitation

In the crude analysis, a 1-year increase in age at injury corresponded to an estimated 0.5-point (95% CI = 0.02 to 1.1) reduction in fear of reinjury at the 3-month follow-up assessment (Tab. 3). The 95% CIs surrounding this estimate included 0 in the adjusted analysis (0.5 [95% CI = −0.1 to 1.0]). Greater self-efficacy at baseline was associated with reduced fear of reinjury at the 12-month follow-up assessment in the crude (1.6 [95% CI = 0.0 to 3.3]) and adjusted (1.7 [95% CI = −0.0 to 3.5]) analyses. No other prognostic factors were identified for fear of reinjury at 3 or 12 months following ACL injury managed with rehabilitation (Tab. 3). Together, all variables accounted for an estimated 6% of the variability in fear of reinjury scores at 3 months and 7% of the variability in fear of reinjury scores at 12 months following ACL injury (Tab. 3).

**Table 3:** Prognostic Factors for Fear of Reinjury at 3 and 12 Months After ACL Injury Managed With Rehabilitation^a^

**Parameter**	**3 mo After ACL Injury (n = 171)**		**12 mo After ACL Injury (n = 82)**	
	**Crude Coefficient (95% CI)**	**Adjusted Coefficient (95% CI)**	**Crude Coefficient (95% CI)**	**Adjusted Coefficient (95% CI)**
Age at injury	0.5 (0.02 to 1.1)*^b^*	0.5 (−0.1 to 1.0)	0.1 (−0.8 to 1.0)	−0.1 (−1.1 to 0.8)
Female sex*^c^*	−3.6 (−11.4 to 4.1)	−2.8 (−10.8 to 5.1)	−2.5 (−15.0 to 10.1)	−4.4 (−19.0 to 10.2)
Lower preinjury activity*^d^*	−3.1 (−10.8 to 4.7)	−1.1 (−9.7 to 7.4)	−4.6 (−17.0 to 7.9)	−5.9 (−21.4 to 9.6)
Baseline knee function*^e^*	0.2 (−0.04 to 0.4)	0.1 (−0.1 to 0.3)	−0.1 (−0.4 to 0.3)	0.0 (−0.3 to 0.3)
Baseline general self-efficacy*^f^*	0.7 (−0.3 to 1.7)	0.6 (−0.4 to 1.6)	1.6 (0.0 to 3.3)*^b^*	1.7 (0.0 to 3.5)*^b^*
Expected recovery at ≤12 mo*^g^*	−5.7 (−16.1 to 4.8)	−3.7 (−14.3 to 7.0)	−8.8 (−31.0 to 13.5)	−9.0 (−33.2 to 15.2)
*R* ^2^		0.06		0.07

^*a*^ACL = anterior cruciate ligament.

^*b*^ *P* ≤ .05.

^*c*^Reference group means male sex.

^*d*^Lower preinjury activity level represents Tegner Activity Scale scores of 0 to 6. Reference group represents higher preinjury activity level; Tegner Activity Scale scores of 7 to 10.

^*e*^Baseline self-reported knee function was assessed with the Single Assessment Numeric Evaluation, with scores ranging from 0 (worst) to 100 (best).

^*f*^Baseline general self-efficacy was assessed with the General Self Efficacy Scale, with scores ranging from 0 to 40 (highest self-efficacy).

^*g*^Reference group means more than 12 months/my knee will never recover.

### Prognostic Factors for Fear of Reinjury at 3 and 12 Months After ACLR

Female sex was a prognostic factor for greater fear of reinjury at 3 months following ACLR in the crude and adjusted analyses (Tab. 4). In the adjusted analysis, female sex was associated with an estimated 15-point (−15 [95% CI = −26 to −3]) greater fear of reinjury at the 3-month follow-up assessment than male sex. Better baseline self-reported knee function was a prognostic factor for reduced fear of reinjury at 12 months following ACLR in the crude and adjusted analyses (Tab. 4). In the adjusted analysis, a 1-point increase on the SANE (range = 0 [worst] to 100 [best]) was associated with an estimated 0.3-point (95% CI = 0.0 to 0.7) reduction in fear of reinjury at the 12-month follow-up assessment. Collectively, all variables accounted for an estimated 8% of the variability in fear of reinjury at 3 months and 11% of the variability in fear of reinjury at 12 months after ACLR (Tab. 4).

**Table 4:** Prognostic Factors for Fear of Reinjury at 3 and 12 Months After ACLR^a^

**Parameter**	**3 mo After ACLR (n = 102)**		**12 mo After ACLR (n = 92)**	
	**Crude Coefficient (95% CI)**	**Adjusted Coefficient (95% CI)**	**Crude Coefficient (95% CI)**	**Adjusted Coefficient (95% CI)**
Age at injury	0.2 (−0.6 to 0.7)	0.2 (−0.6 to 1.1)	−0.7 (−1.7 to 0.2)	−0.6 (−1.6 to 0.5)
Female sex*^b^*	−15.1 (−26.2 to −4.0)*^c^*	−14.5 (−25.9 to −3.1)*^c^*	−7.1 (−20.2 to 5.9)	−6.9 (−19.8 to 6.0)
Lower preinjury activity*^d^*	4.0 (−8.8 to 16.7)	2.7 (−10.5 to 15.9)	9.0 (−6.1 to 24.0)	1.6 (−15.0 to 18.1)
Baseline knee function*^e^*	0.1 (−0.1 to 0.4)	0.1 (−0.1 to 0.4)	0.4 (0.1 to 0.7)*^f^*	0.3 (0.01 to 0.7)*^f^*
Baseline general self-efficacy*^g^*	0.4 (−0.9 to 1.7)	0.2 (−1.2 to 1.5)	0.3 (−1.1 to 1.8)	0.3 (−1.2 to 1.7)
Expected recovery at ≤12 mo*^h^*	−4.3 (−20.3 to 11.6)	−2.0 (−17.6 to 13.6)	−11.3 (−28.3 to 5.7)	−9.9 (−26.8 to 7.0)
*R* ^2^		0.08		0.11

^*a*^ACLR = anterior cruciate ligament reconstruction.

^*b*^Reference group mean male sex.

^*c*^ *P* ≤ .01.

^*d*^Lower preinjury activity level represent Tegner Activity Scale scores of 0 to 6. Reference group represent higher preinjury activity level; Tegner Activity Scale scores of 7 to 10.

^*e*^Baseline self-reported knee function was assessed with the Single Assessment Numeric Evaluation, with scores ranging from 0 (worst) to 100 (best).

^*f*^ *P* ≤ .05.

^*g*^General self-efficacy was assessed with the General Self Efficacy Scale, with scores ranging from 0 to 40 (highest self-efficacy).

^*h*^Reference group means more than 12 months/my knee will never recover.

## Discussion

A high degree of fear of reinjury was common following ACL injury managed with rehabilitation alone or ACLR. Additionally, the certainty of not having a new knee reinjury was low at the 3-, 6-, and 12-month follow-up assessments irrespective of management strategy. Fear of reinjury decreased between 3 and 12 months after injury for patients who were treated with rehabilitation (and had no ACLR within 24 months of injury). However, this was not observed in patients who later underwent an ACLR who reported worse fear of reinjury at 3 and 12 months after injury compared with those who did not proceed to ACLR. Following ACLR, patients’ fear of reinjury decreased between the 3- and 12-month follow-up assessments. These findings were consistent irrespective of whether fear of reinjury was assessed with ACL-QOL item 31 or ACL-RSI item 9.

Different prognostic factors for fear of reinjury were identified for patients treated with rehabilitation and those who underwent ACLR. Older age at injury was related to reduced fear of reinjury 3 months after ACL injury managed with rehabilitation. Greater self-efficacy at baseline was associated with reduced fear of reinjury at the 12-month follow-up assessment in patients not treated with ACLR. In contrast, female sex was related to more fear of reinjury at 3 months postoperatively, and better baseline knee function was related to reduced fear of reinjury at 12 months postoperatively in those who underwent ACLR.

### Fear and Certainty of Reinjury

We found that fear of reinjury scores were similar on average between people treated with rehabilitation alone and those treated with ACLR. However, patients who were initially treated with rehabilitation but later underwent ACLR reported higher fear of reinjury at 3 and 12 months after injury compared with patients who did not undergo ACLR. We have previously reported that the most common reason for not having ACLR in our cohort was no knee instability and no problems with knee function.^45^ So it is not surprising that patients who decided not to have ACLR reported less fear of reinjury. Knee-related confidence and functional performance on a hop test at the time of return to sport have been associated with an increased risk of a second ACL injury after ACLR.^46^ Further investigations are needed to determine whether patients with high fear of reinjury before ACLR experienced improvement in function and reductions in fear of reinjury after ACLR.

Although fear of reinjury scores improved over time, most patients experienced fear of reinjury at the 12-month follow-up assessment, when care has typically ceased and patients have generally returned to desired physical activities. Considering as many as 1 in 3 people who return to sport after ACLR experience a new knee injury,^8^^,^^47^ this may reflect a realistic understanding of this risk. This suggests that these fears may be warranted, and people may be somewhat accurate in anticipating their own likelihood of reinjury.^48^ The contributing factors to an increased risk of reinjury in those fearing reinjury are not yet understood. Potential contributing factors include impaired knee function combined with returning to pivoting or contact sports resulting in a greater risk of reinjury.

The high prevalence of fear of reinjury in our study highlights the importance of evaluating fear of reinjury throughout rehabilitation and, when present, warrants further assessment of physical and psychological function. Not only is fear of reinjury related to the decision not to return to sport, but fear of reinjury can also prevent someone participating in exercise and other forms of physical activity.^48^^,^^49^^,^^50^ Addressing fear of reinjury and negotiating decisions around sport and physical activity participation can have important implications for long-term quality of life after ACL injury.^11^ Addressing psychological barriers to resuming activity participation is a recommended objective of ACL management.^1^ Strategies that may be used in attempt to reduce fear of reinjury include referral to a psychologist,^1^ a relatable role model, relaxation, goal-setting, and mental practice.^51^ Additionally, we recommend that an individual with fear of reinjury undergoes a comprehensive assessment of knee function and that any functional deficits are restored prior to returning to sport or other activities with an increased risk of reinjury.

### Prognostic Factors and Management Approach

We found that greater general self-efficacy at baseline was a prognostic factor for reduced fear of reinjury at 12 months following rehabilitation but not following ACLR. Although knee self-efficacy typically increases during rehabilitation after ACL injury,^52^ our results suggest people with low self-efficacy at baseline may experience greater fear of reinjury throughout rehabilitation. The General Self Efficacy Scale assesses the strength of an individual’s belief in his or her own ability to respond to novel or difficult situations and to deal with any associated obstacles or setbacks.^41^ Greater self-efficacy has been associated with greater rehabilitation adherence following musculoskeletal injury.^53^ Additionally, internal locus of control is an important determinant of self-efficacy in patients with an ACL injury.^54^ A high internal locus of control is associated with greater satisfaction with knee function compared with a more external locus of control.^55^ Dissatisfaction with knee function was one of the main reasons patients in our study decided to undergo ACLR.^45^ It is possible that patients with high general self-efficacy, trusting of their own ability to solve problems, are better suited to adapt to ACL injury and achieve favorable outcomes with nonsurgical ACL management. People with lower general self-efficacy and higher external locus of control are more reliant on the influence of powerful others, in our case an orthopedic surgeon, which may make them more likely to choose to undergo ACLR. However, it is not clear whether these individuals would have experienced better outcomes and reduced fear of reinjury following management with ACLR.

We found that female sex was related to worse fear of reinjury following ACLR, but not following management with rehabilitation alone. It is possible that women were more likely to fear additional surgery associated with reinjury or the postoperative pain and challenges that follow ACL surgery. A qualitative study found that fear of undergoing surgery and the recovery process again was a key contributor to fear of reinjury after ACLR.^18^ This was common in people who reported a painful surgery, prolonged rehabilitation, and the inconvenience of functional restrictions.^18^ Women have been found to have a higher prevalence of phobias and greater levels of fear related to specific objects and situations.^56^ Additionally, women were found to be more sensitive to pain,^57^ have a lower pain threshold,^58^ report greater pain-related disability,^59^ and report a greater fear of pain^60^ than men. In people with low back pain, higher pain intensity was associated with higher fear of reinjury in women but not in men.^61^ Thus, there may be specific factors to do with ACL surgery that place women at greater risk of fear of reinjury than their male counterparts. Further research is needed to understand these findings and to improve understanding of the characteristics of patients who benefit from a nonsurgical approach to ACL injury management.

We found that better self-reported knee function at baseline was a prognostic factor for reduced fear of reinjury at 12 months following ACLR but not following management with rehabilitation alone. In the KANON Trial, worse knee symptoms and function were identified as a prognostic factor for worse 5-year pain and function following management with early ACLR (but not following management with rehabilitation alone).^62^ Improving knee function prior to undergoing ACLR through prehabilitation has been associated with higher return to sport rates and better postoperative knee function.^63^ Higher return to sport rates and better postoperative knee function are associated with higher knee self-efficacy^64^ and reduced fear of reinjury.^65^ Additionally, greater preoperative pain has been associated with increased kinesiophobia at 3 months following ACLR.^23^ Further investigations are needed to determine whether improving preoperative knee function has a positive impact on postoperative fear of reinjury.

### Limitations

There is no established outcome measure to evaluate fear of reinjury that is valid and reliable for use in ACL injured individuals. The Tampa Scale of Kinesiophobia is the most common outcome measure used to evaluate fear of reinjury after ACLR.^66^ However, this questionnaire was initially developed to assess fear of movement and reinjury in people with chronic pain, and the specific questions may not be relevant for people with ACL injury. Therefore, we evaluated fear of reinjury with an ACL-QOL item and reported outcomes for 2 related constructs to provide a more detailed picture of fear of reinjury in this sample. Our findings suggest that K-SES item D2 may be particularly useful to measure at baseline because scores were markedly different from the ACL-QOL and ACLRSI items. Although the measures comprising these items are valid and reliable for use in populations with ACL injury, the psychometric properties for these stand-alone items have not been evaluated.

Additionally, not all patients completed outcomes at each follow-up point. It is possible that the nonresponders experienced more fear of reinjury than those who completed follow-up assessments, impacting the generalizability of results. The purpose of the NACOX Study was to establish a “real-world” cohort of patients with ACL injury who receive usual care reflecting current practice in Sweden. As a result, these findings are generalizable to individuals with ACL injury in Sweden. However, a limitation of this design is that rehabilitation strategies were not standardized, and we were not able to evaluate the impact of specific rehabilitation protocols on patient outcomes. To improve generalizability of findings, patients with a history of contralateral ACL injury were included in the study. It is possible that previous experience of ACL injury could influence reinjury fears. However, a similar proportion of individuals treated with rehabilitation alone (7% at 3 months, 6% at 12 months) and ACLR (7% at 3 months, 8% at 12 months) had a history of contralateral injury, so we would not expect this to influence our study findings. Additionally, there is potential for unmeasured confounders (eg, episodes of giving way or subsequent injury, depression or anxiety, social support) to bias effect estimates.

## Clinical Trial Registration

This study is an exploratory analysis of data from the Natural Corollaries and Recovery After ACL-injury (NACOX) Study (NCT02931084).
